# Trait conscientiousness and the personality meta-trait stability are associated with regional white matter microstructure

**DOI:** 10.1093/scan/nsw037

**Published:** 2016-03-24

**Authors:** Gary J. Lewis, Simon R. Cox, Tom Booth, Susana Muñoz Maniega, Natalie A. Royle, Maria Valdés Hernández, Joanna M. Wardlaw, Mark E. Bastin, Ian J. Deary

**Affiliations:** ^1^Department of Psychology, Royal Holloway, University of London, Egham, Surrey TW20 0EX, UK; ^2^Department of Psychology; ^3^Centre for Cognitive Ageing and Cognitive Epidemiology, University of Edinburgh, 7 George Square, Edinburgh EH8 9JZ, UK; ^4^Scottish Imaging Network, a Platform for Scientific Excellence (SINAPSE) Collaboration, Edinburgh, UK; ^5^Brain Research Imaging Centre, Neuroimaging Sciences, Centre for Clinical Brain Sciences, University of Edinburgh, Western General Hospital, Edinburgh EH4 2XU, UK

**Keywords:** personality, conscientiousness, stability, white matter microstructure, fractional anisotropy, uncinate fasciculus

## Abstract

Establishing the neural bases of individual differences in personality has been an enduring topic of interest. However, while a growing literature has sought to characterize grey matter correlates of personality traits, little attention to date has been focused on regional white matter correlates of personality, especially for the personality traits agreeableness, conscientiousness and openness. To rectify this gap in knowledge we used a large sample (*n* > 550) of older adults who provided data on both personality (International Personality Item Pool) and white matter tract-specific fractional anisotropy (FA) from diffusion tensor MRI. Results indicated that conscientiousness was associated with greater FA in the left uncinate fasciculus (β = 0.17, *P* < 0.001). We also examined links between FA and the personality meta-trait ‘stability’, which is defined as the common variance underlying agreeableness, conscientiousness, and neuroticism/emotional stability. We observed an association between left uncinate fasciculus FA and stability (β* *= 0.27, P < 0.001), which fully accounted for the link between left uncinate fasciculus FA and conscientiousness. In sum, these results provide novel evidence for links between regional white matter microstructure and key traits of human personality, specifically conscientiousness and the meta-trait, stability. Future research is recommended to replicate and address the causal directions of these associations.

## Introduction

Understanding the origins of individual differences in personality traits has been an enduring challenge for psychologists (Eysenck, 1967; Gray and McNaughton, 2000; Zuckerman, 2005). An area of current interest is the association between brain white matter microstructure and personality traits. Research has shown links between white matter microstructure and both neuroticism and (to a lesser extent) extraversion (Bjørnebekk *et al.*, 2013; McIntosh *et al.*, 2013). Less well studied are the other Big Five personality dimensions: agreeableness, conscientiousness and openness. This relative lack of attention probably reflects the clinical orientation of much of the work in this field to date (e.g. Westlye *et al.*, 2011; McIntosh *et al.*, 2013), in line with neuroticism and extraversion (and closely related constructs) showing strong associations with psychopathology and well-being (Kotov *et al.*, 2010). However, agreeableness, conscientiousness and openness are important traits in their own right, with links to a range of important life outcomes (McCrae, 1996; Bogg and Roberts, 2004; Graziano and Tobin, 2013), and therefore deserve further investigation.

To address this gap in knowledge, we sought to advance understanding regarding links between white matter microstructure and agreeableness, conscientiousness, and openness using a large sample of older adults drawn from the Lothian Birth Cohort 1936 (LBC1936) who completed personality measures and underwent brain MRI scanning. To capture white matter microstructure we employed fractional anisotropy (FA) measured in specific tracts of interest using diffusion tensor MRI (DT-MRI) and quantitative tractography. We next briefly summarize research addressing regional white matter microstructure links with personality, before moving to tests of association between white matter microstructure and agreeableness, conscientiousnessand openness in the LBC1936.

### Personality and brain white matter microstructure: a brief overview

Broad consensus has now been reached that much variation in human personality can be understood through five dimensions, commonly referred to as the Big Five (John *et al.*, 2008): agreeableness, conscientiousness, extraversion, neuroticism and openness. Because there are correlations among the five traits, two meta-traits are proposed to sit above these five dimensions (Digman, 1997; DeYoung, 2006) and a larger number of facets of each of the dimensions sit at a level below in this hierarchy of traits (DeYoung *et al.*, 2007). Although interest in the neural bases of personality traits precedes current thinking regarding personality trait organization (e.g. Eysenck, 1967), modern neuroimaging technologies, coupled with the broad-based acceptance concerning the importance of the Big Five as a core model of personality differences, has led to an increase in efforts directed towards understanding the neurostructural foundations of these traits (DeYoung and Gray, 2009).

Most recently this interest has turned towards examining brain white matter microstructure correlates of personality, with FA and mean diffusivity (MD) the most common measures reported. Higher levels of FA and lower levels of MD are typically indicative of better white matter microstructure (with the exception of conditions such as abnormal hypermyelination: e.g. Moritani *et al.*, 2008). There are reported associations between neuroticism and white matter microstructure, with links to the uncinate fasciculus—a tract connecting the anterior temporal lobe with the orbitofrontal cortex (Catani *et al.*, 2002)—being particularly notable. For example, McIntosh *et al.* (2013), in the sample of healthy Scottish elderly adults (*n* > 550) who are also the participants in this study, observed that FA in the left uncinate fasciculus was negatively associated with neuroticism (β = −0.12). Similarly, in a sample of healthy, adult Norwegians (*n* = 265), widespread negative associations in FA and positive associations with MD, including the uncinate fasciculus, have been reported with neuroticism (Bjørnebekk *et al.*, 2013). Further, using a sample of 51 healthy adults, neuroticism was positively associated (*r* = 0.59) with MD in the left uncinate fasciculus: while no significant association with FA was found, a moderate negative association was observed with the left uncinate fasciculus (*r* = −0.29: Xu and Potenza, 2012). Finally, harm avoidance, which is in turn highly correlated with neuroticism (e.g. Jokela and Keltikangas-Järvinen, 2011), has also shown links with FA in the right uncinate fasciculus (*r* = −0.71) in a small sample of Italian adolescents (*n* = 20; Taddei *et al.*, 2012).

For other Big Five traits the results are less consistent. McIntosh *et al.* (2013), in addition to the observation of a link between left uncinate fasciculus FA and neuroticism noted earlier, reported a positive association with right uncinate fasciculus FA and extraversion (β = 0.13). Xu and Potenza (2012) reported that openness and agreeableness were positively related to FA (*r*s from 0.14 to 0.47) and negatively with MD (*r*s from −0.62 to −0.58) in the corona radiata and superior longitudinal fasciculus (bilaterally), but observed no links with FA or MD for extraversion or conscientiousness. In contrast, in a sample of university students (*n* = 72), Jung *et al.* (2010) reported that openness was negatively associated with FA in the right uncinate fasciculus and anterior thalamic radiation, but no associations were reported for the other Big Five traits. Of additional note, Bjørnebekk *et al.* (2013), in a sample of 265 healthy adults, reported no white-matter associations for Big Five traits other than neuroticism (as detailed earlier).

More broadly, indirect evidence for associations between personality and white matter microstructure comes from studies of groups with personality disorders. Carrasco *et al.* (2012) compared FA in individuals with a borderline personality disorder (*n* = 28)—a diagnosis linked with low agreeableness, low conscientiousness and neuroticism (Widiger and Costa, 1994)—to FA in a healthy control group (*n* = 26). Those with a borderline diagnosis showed lower FA in the left genu and rostral corpus callosum and in left prefrontal fasciculi, including the anterior thalamic radiation. Sundram et al. (2012) reported that antisocial personality disorder (*n* = 30: 15 cases, 15 controls)—which in turn is linked to low agreeableness and low conscientiousness (Lynam and Widiger, 2001)—was negatively associated with FA in several regions, including the genu of the corpus callosum and the right uncinate fasciculus. Finally, Mao *et al.* (2011) reported that psychoticism measured using the Eysenck Personality Questionnaire—which is noted to be a combination of agreeableness and conscientiousness (e.g. McCrae and Costa, 1985)—was related to FA in the arcuate fasciculus (*r* = −0.29, −0.36: L/R hemisphere, respectively) in a sample of patients with epilepsy (*n* = 65); no significant correlations with neuroticism or extraversion were observed.

### The current study

Beyond neuroticism, relatively little is known regarding brain white matter microstructure associations with Big Five dimensions of personality. This observation reflects the fact that only a handful of studies in this field have been reported to date. Moreover, most of these studies have been confined to small samples (i.e. *n* < 80, with the exceptions of Bjørnebekk *et al.*, 2013, McIntosh *et al.*, 2013). Here we sought to address this gap in knowledge. As noted earlier, recent work with the LBC1936 has already examined white matter microstructure links to neuroticism and extraversion (McIntosh *et al.*, 2013): accordingly, here we specifically focused on the traits of agreeableness, conscientiousness and openness. Although results in the field have been mixed, we made the following predictions based on the reported associations between these three traits and white matter microstructure. We hypothesized that there would be positive links from the corpus callosum genu and uncinate fasciculus to both agreeableness and conscientiousness in line with observations in related constructs (Carrasco *et al.*, 2012; Sundram *et al.*, 2012). We also predicted negative links from the uncinate fasciculus and anterior thalamic radiation to openness, in line with findings from Jung *et al.* (2010). All other tract-trait analyses were deemed exploratory.

## Methods

### Participants

The LBC1936 is a sample (*n* = 1091) of community-dwelling and relatively healthy individuals without dementia who, at the time of being recruited in older age, resided in or close to the city of Edinburgh (the Lothian region), Scotland. All were born in 1936. Most took part in the Scottish Mental Survey of 1947 in which they undertook a mental ability test at school on 4 June 1947. They were recruited in older age, at about mean age 70 years, to Wave 1 of a study of cognitive ageing. They have been extensively followed-up every 3 years during their 70s. Full details of the study are available in the form of a study protocol (Deary *et al.*, 2007), cohort profile (Deary *et al.*, 2012), and brain imaging protocol (Wardlaw *et al.*, 2011). The data from the LBC1936 cohort used in this study were obtained at Wave 2 of the study, when the participants had a mean age of about 73 years. This was the first wave in which brain imaging was performed. Participants travelled to the Wellcome Trust Clinical Research Facility in Edinburgh for testing. The study was approved by the Lothian (REC 07/MRE00/58) and Scottish Multicentre (MREC/01/0/56) Research Ethics Committees and all subjects gave written informed consent.

## Measures

### Personality

Big Five personality traits were measured using the well-characterized and validated International Personality Item Pool (IPIP) 50-item inventory (Goldberg, 1999; Gow *et al.*, 2005), with 10 items each tapping emotional stability (the opposite of neuroticism), extraversion, intellect (similar to openness), agreeableness and conscientiousness. Participants rated each self-description on a 1 (very inaccurate) to 5 (very accurate) scale.

### History of stroke, hypertension, and cigarette use

At Wave 2, participants gave self-reported information on history of stroke, hypertension and cigarette use as part of a medical interview. We used these as covariates in our analyses, as all are linked to brain white matter microstructure damage (e.g. Gons *et al.*, 2011; Staals *et al.*, 2014; Wardlaw *et al.*, 2014). All were coded as a binary variable. Stroke was coded as 0 = no self-report of stroke, and 1 = self-report of stroke. Hypertension was coded as 0 = no, and 1 = yes. Cigarette use was coded as 0 = never smoked, and 1 = current or past smoker.

### Brain white matter tract microstructure

Diffusion MRI was performed using a GE Signa HDXt 1.5-T clinical scanner (General Electric, USA) using a self-shielding gradient set with maximum gradient strength of 33 mT m^−1^, and a manufacturer-supplied eight-channel phased-array head coil. Single-shot, spin-echo echo-planar imaging diffusion-weighted volumes (*b *=* *1000 s mm^−2^) were acquired in 64 non-collinear directions, along with seven T_2_-weighted volumes (*b *=* *0 s mm^−2^). Seventy-two contiguous axial slices of thickness 2 mm were acquired with a field of view of 256 × 256 mm and matrix size of 128 × 128, giving a resolution of 2 × 2 × 2 mm. The repetition time was 16.5 s and echo time 95.5 ms. The total scan time was ∼20 min (see Wardlaw *et al.*, 2011 for details of the full imaging protocol).

Diffusion MRI data were converted into NIfTI (http://nifti.nimh.nih.gov/nifti-1) format. Pre-processing steps were performed using the tools provided in FSL (FMRIB, Oxford, UK; www.fmrib.ox.ac.uk) to extract the brain, remove bulk motion and eddy current induced artifacts and estimate water DT parameters. Brain connectivity data were created using the BEDPOSTX/ProbTrackX tractography algorithm with a two-fiber model and 5000 streamlines to reconstruct tracts of interest.

An automatic tract selection method—probabilistic neighbourhood tractography (PNT)—with good reproducibility (Clayden *et al.*, 2009a), based on a model of tract topology (Clayden *et al.*, 2007), was used to generate equivalent tracts of interest in each participant. PNT, which was implemented in the TractoR package (http://www.tractor-mri.org.uk), optimizes the choice of seed point for tractography by estimating the best matching tract from a series of candidate tracts generated from a neighbourhood of voxels (7 × 7 × 7) placed around a voxel transferred from standard space against a pre-defined reference tract. The topological tract model was also used to reject any false positive connections (Clayden *et al.*, 2009b). As a consequence, this procedure significantly improved tract segmentation. Twelve white-matter tracts were segmented: the genu and splenium of corpus callosum, cingulum bundles, anterior thalamic radiations, uncinate, arcuate and inferior longitudinal fasciculi. For each subject, the seed point that produced the best match tract to the reference for each of the 12 pathways was determined, with the resulting tractography mask applied to each participant’s FA volume. Tract-averaged FA values were calculated from these masks and used in all subsequent analyses. In order to make sure that the segmented tracts were anatomically plausible representations of the tracts of interest, a researcher (S.M.M.) visually inspected all masks blind to the other study variables and excluded tracts that showed aberrant or truncated pathways.

## Results

Of the 866 LBC1936 participants who attended Wave 2 testing, 668 (males = 53%) individuals provided usable tractography data which passed the visual quality control procedures outlined earlier. The range of available data was from a low of *n* = 556 for the left anterior thalamic radiation to a high of *n* = 664 for the right inferior longitudinal fasciculus.

We first tested for association between white matter microstructure, as assessed by tract-averaged FA, and the traits agreeableness, conscientiousness, and openness. For hypothesized white-matter-personality links, our nominal level for statistical significance was *P* < 0.05. These results are detailed in full in Table 1. For all other associations, we used a false discovery rate (FDR) corrected *P*-value (Benjamini and Hochberg, 1995). These results are detailed in full in Table 2. Additional analyses using alternative measures of the Big Five traits (i.e. NEO-FFI traits measured at age 70, and thus collected 3 years prior to the diffusion MRI, and a mean score for each dimension derived from the NEO-FFI_age70_ and IPIP_age73_) are reported in the Supplementary Materials. These subsidiary results are mostly unchanged from those reported below, with all key results essentially identical.

**Table 1.** Correlations between white-matter FA and personality traits (agreeableness, conscientiousness and openness) for the hypothesized links

	Agreeableness		Conscientiousness		Openness	
	*r*	*P*	*r*	*P*	*r*	*P*
Corpus callosum genu	0.03	0.41	0.03	0.41		
L uncinate fasciculus	0.13	**0.002**	0.17	**0.00005**	0.07	0.10
R uncinate fasciculus	0.05	0.19	0.09	**0.03**	0.05	0.23
L anterior thalamic radiation					0.06	0.17
R anterior thalamic radiation					0.09	**0.03**

Note: Uncorrected *P*-values for all correlations; L,  left; R, right; significant *P*-values are bolded; blank cells denote that associations were not hypothesized a priori. Exploratory results are reported in Table 2.

**Table 2.** Correlations between white-matter FA and personality traits (agreeableness, conscientiousness and openness) for the exploratory analyses (FDR-corrected *P*-values)

	Agreeableness		Conscientiousness		Openness	
	*r*	*P*	*r*	*P*	*r*	*P*
Corpus callosum genu					−0.02	0.72
Corpus callosum splenium	0.09	0.10	0.06	0.26	0.02	0.72
L anterior thalamic radiation	0.05	0.37	0.06	0.28		
R anterior thalamic radiation	0.03	0.65	0.12	**0.01**		
L cingulum	0.06	0.28	0.13	**0.009**	0.03	0.59
R cingulum	0.05	0.37	0.14	**0.008**	0.06	0.28
L arcuate fasciculus	0.04	0.45	0.12	**0.02**	−0.02	0.72
R arcuate fasciculus	0.02	0.75	0.06	0.28	0.01	0.82
L inf. longitudinal fasciculus	0.10	**0.03**	0.10	**0.03**	0.06	0.28
R inf. longitudinal fasciculus	0.07	0.20	0.13	**0.009**	−0.01	0.89

Note: FDR corrected *P*-values for all correlations; L, left; R, right; inf., inferior; significant *P*-values are bolded; blank cells denote that associations were hypothesized a priori. These results are reported in Table 1.

As predicted, we observed significant positive associations between uncinate fasciculus FA bilaterally with conscientiousness (*r* = 0.17/.09; *P* = 0.00005/.03, L/R hemisphere, respectively: also see Supplementary Figure S1), left uncinate fasciculus FA with agreeableness (*r* = 0.13; *P* = 0.002), and right anterior thalamic radiation FA with openness (*r* = 0.09; *P* = 0.03).

**Fig. 1.**
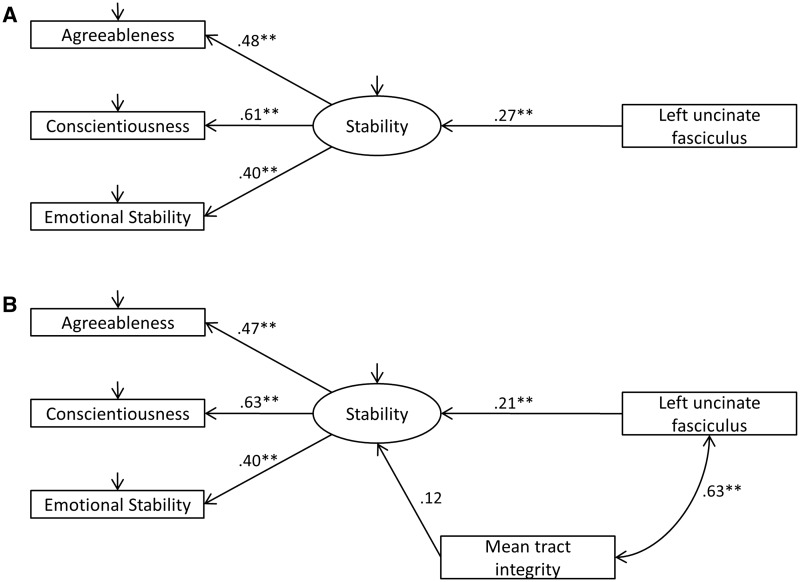
Structural equation model test of association between meta-trait stability and left uncinate fasciculus FA (Panel A without controlling for mean tract integrity, Panel B controlling for mean tract integrity).Note: Model fit statistics (Panel 1A/1B, respectively): χ^2 ^=^ ^0.02/1.27, df = 2/4, *P* = 0.99/0.87; RMSEA = 0.00/0.00; CFI = 1.0/1.0; all path values are standardized coefficients; *P < 0.05, **P < 0.01; the variance of the latent factor for stability was constrained to 1; model estimation was performed using full information maximum-likelihood; emotional stability reflects the opposite of neuroticism.

Our exploratory analyses revealed significant associations between conscientiousness and FA measured in bilateral cingulum (*r* = 0.13/.14; *P* = 0.009/0.008, L/R hemisphere, respectively), inferior longitudinal fasciculus (*r* = 0.10; *P* = 0.03; *r* = 0.13; *P* = 0.009, L/R hemisphere, respectively), left arcuate fasciculus (*r* = 0.12; *P* = 0.02) and right anterior thalamic radiation (*r* = 0.12; *P*  = 0.01). Agreeableness was significantly associated with FA in left inferior longitudinal fasciculus (*r* = 0.10; *P* = 0.03).

In order to establish whether the observed associations were specific to individual tracts, or reflected more generalized tract integrity (see Booth *et al.*, 2014), we next examined whether the above associations were robust when controlling for mean tract integrity. To this end we took a mean score of tract integrity, excluding the tract of interest for any given analysis, and used this score as a control variable. Not all individuals had usable data for each of the 12 tracts; as such, we used only individuals who had 10 or more of the tracts available for analysis. Left uncinate fasciculus (β = 0.12, *P* = 0.03) remained an independent predictor of conscientiousness when controlling for mean tract integrity (left uncinate fasciculus was correlated with mean tract integrity *r* = 0.62, *P* < 0.001). Moreover, this association was almost unchanged when additionally controlling for history of stroke, hypertension, and smoking. However, the other significant associations between FA and personality fell below nominal significance when controlling for mean tract integrity. Mean tract integrity was associated with agreeableness (*r* = 0.10, *P* = 0.01) and conscientiousness *r* = 0.18, *P* < 0.001, but not with openness *r* = 0.06, *P* = 0.12) (see also Booth *et al.*, 2014). Finally, in line with the association between openness and general intelligence (Ashton *et al.*, 2000) we also examined whether openness was associated with white-matter microstructure when controlling for general intelligence (defined here as the first unrotated principle component from six WAIS III tests: this component accounted for 51% of the test score variance); however, the pattern of correlations was virtually unchanged to those reported earlier and no significant observations were noted.

The positive link between left uncinate fasciculus FA and both conscientiousness and agreeableness (albeit with the latter not significant when controlling for mean tract integrity), alongside an association with neuroticism as reported in previous work in this sample (McIntosh *et al.*, 2013), gives rise to the possibility that the personality link with this tract is better represented at the meta-trait level. Specifically, a body of work has indicated that covariation between agreeableness, conscientiousness and neuroticism can be understood as a higher-order latent factor commonly termed ‘stability’ (DeYoung, 2006; also see Digman, 1997). To test for this possibility we used a structural equation modelling approach using Amos version 22 (Arbuckle, 2013). This analysis indicated that the higher-order latent factor of stability was significantly associated with left uncinate fasciculus FA (β = 0.27, *P* < 0.001: see Figure 1, Panel A). This association remained significant (β = 0.21, *P* < 0.001) when controlling for mean tract integrity (see Figure 1, Panel B: note, neuroticism/emotional stability and mean tract integrity were correlated *r* = −0.09, *P* = 0 .02). We also tested whether variation in agreeableness, conscientiousness, and neuroticism/emotional stability (i.e. the residual variance for each of these traits), over and above the stability factor, showed links to left uncinate fasciculus FA. No such links were observed (all βs < ± 0.02, all *P*s > 0.88) indicating that left uncinate fasciculus’s link to personality is most parsimoniously understood at the meta-trait level.

## Discussion

This study used a relatively large sample (*n* > 550) of older adults to examine links between regional white matter microstructure and several core dimensions of personality: specifically, agreeableness, conscientiousness and openness. Several key results were noted. First, we observed a positive link between left uncinate fasciculus FA and conscientiousness. We also noted a positive association between left uncinate fasciculus FA and agreeableness (although this association fell below nominal significance when controlling for mean tract integrity). These associations, coupled with recent work highlighting a negative link between neuroticism and left uncinate fasciculus FA (McIntosh *et al.*, 2013), led us to examine whether the common factor accounting for shared variance in agreeableness, conscientiousness and neuroticism/emotional stability—stability (DeYoung, 2006; also see Digman, 1997)—was associated with left uncinate fasciculus microstructure. This additional analysis highlighted the fact that the personality meta-trait of stability was the most parsimonious level at which to account for associations between individual personality traits and the left uncinate fasciculus; i.e. the associations between this tract and agreeableness, conscientiousness or neuroticism/emotional stability were mediated by stability.

Is this association between stability and the left uncinate fasciculus plausible? Stability is argued to reflect the ‘need to maintain a stable organization of psychosocial function’ (DeYoung, 2006:1149) and thus would likely be dependent on neural systems instantiating impulse control, emotion regulation and related cognitive-affective processes. The uncinate fasciculus is a major connection between anterior temporal and ventral prefrontal regions of the brain. Social-cognitive abilities are associated with both orbitofrontal and anterior temporal functioning (MacPherson *et al.*, 2015; Olson *et al.*, 2013), diffusion characteristics in left uncinate fasciculus (Jalbrzikowski *et al.*, 2014) and with neuroticism and agreeableness (e.g. Nettle and Liddle, 2008); thus the uncinate fasciculus appears to be ideally placed to modulate these personality traits and related emotional processes (Cardinal *et al.*, 2002).

Specific recommendations for future research are warranted. First, we were only able to explain a modest proportion of personality trait variation via the white matter tract microstructure measures examined here, and previous research (e.g. Xu and Potenza, 2012) has identified white matter microstructure links to personality in tracts not investigated in this study. The modest effects observed here thus likely reflect the fact that multiple brain regions and neuroanatomical components (e.g. white matter microstructure, regional cortical thickness) collectively account for individual differences in personality. Future work, then, should seek to extend on the scope of the current observations in order to more fully characterize the neuroanatomical bases of personality. Second, with the cross-sectional design employed here we are not able to determine the direction of causality underlying the observed relationships between white matter microstructure and personality. In this sample of older adults it is conceivable that individual differences in brain structure personality lead to personality trait variation, or that personality leads to changes over time in brain structure, or both. The latter perspective is somewhat mitigated by our key results robust when controlling for the effects of mean tract integrity. Nonetheless, future work utilizing longitudinal studies of brain development and change could contribute to understanding the direction of causal influences underlying such associations (Hedman *et al.*, 2012).

In summary, we provide evidence from a relatively large sample of older adults that core aspects of personality—specifically conscientiousness and the meta-trait of stability—are associated with regional white matter microstructure; specifically, left uncinate fasciculus. Future research is recommended in order to establish the causal direction of this association, as well as to characterize more fully the neuroanatomical bases of personality.

## Supplementary Material

Supplementary Data
