# Supplementary material for: Trait conscientiousness and the personality meta-trait stability are associated with regional white matter microstructure
Source: Soc Cogn Affect Neurosci. 2016 Mar 24;11(8):1255–61. doi: 10.1093/scan/nsw037 (PMC4967799; doi:10.1093/scan/nsw037)
Supplement: Supplementary Data [file supp_11_8_1255__index.html]

Trait conscientiousness and the personality meta-trait stability are associated with regional white matter microstructure — Trait conscientiousness and the personality meta-trait stability are associated with regional white matter microstructure — Supplementary Data 

# Trait conscientiousness and the personality meta-trait stability are associated with regional white matter microstructure

## Supplementary Data

files

- Supplementary Data - docx file
