# Supplementary material for: Trait conscientiousness and the personality meta-trait stability are associated with regional white matter microstructure
Source: Soc Cogn Affect Neurosci. 2016 Mar 24;11(8):1255–61. doi: 10.1093/scan/nsw037 (PMC4967799; doi:10.1093/scan/nsw037)
Supplement: Supplementary Data [file supp_nsw037_scan-15-722-File002.docx]

**Supplementary Materials**

Supplementary Figure 1. Scatter plot of the relationship between left uncinate fasciculus fractional anisotropy and conscientiousness.

Note. r = .17, p = .00005

Supplementary Figure 2. Structural equation model test of association between meta-trait stability and left uncinate fasciculus fractional anisotropy (Panel A without controlling for mean tract integrity, Panel B controlling for mean tract integrity).


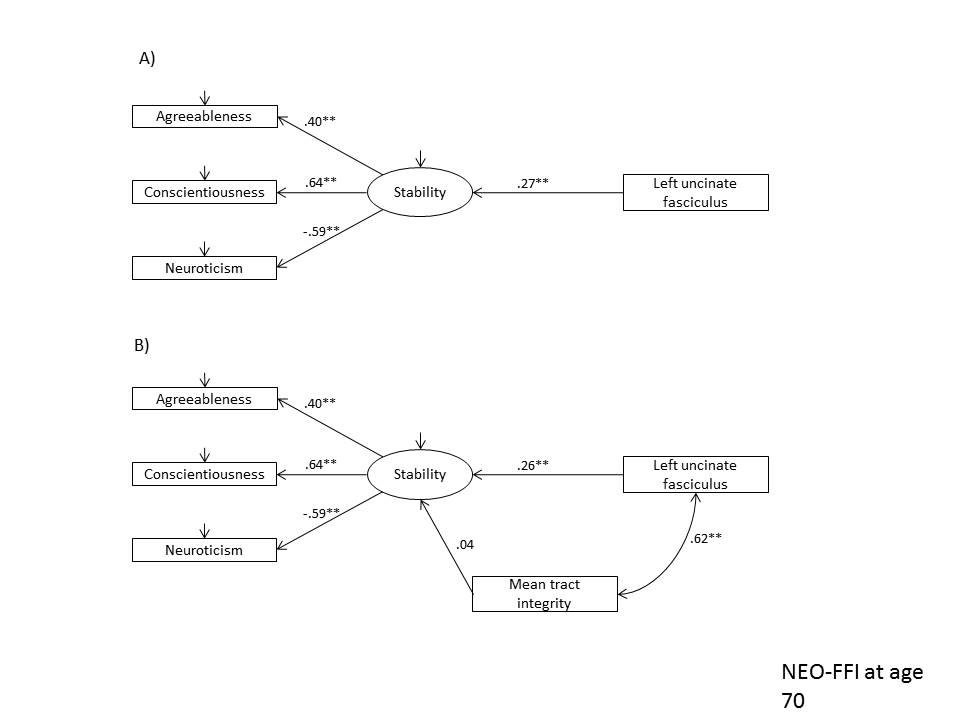


Note. NEO-FFI traits measured at age 70 were used for these analyses.

Supplementary Figure 3. Structural equation model test of association between meta-trait stability and left uncinate fasciculus fractional anisotropy (Panel A without controlling for mean tract integrity, Panel B controlling for mean tract integrity).


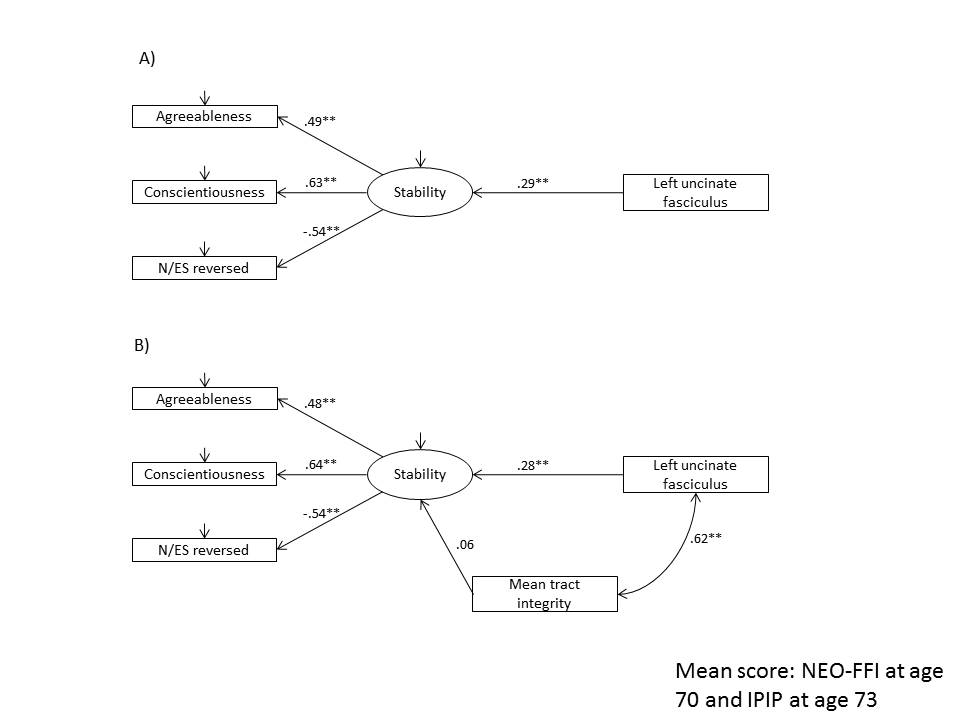


Note. The mean score of NEO-FFI traits measured at age 70 and IPIP traits measured at age 73 were used for these analyses.Supplementary Table 1. Correlations between white-matter fractional anisotropy and personality traits (using the NEO-FFI measured at age 70: agreeableness, conscientiousness, and openness) for the hypothesized links.

|  | Agreeableness | | Conscientiousness | | | Openness | | |  |
| --- | --- | --- | --- | --- | --- | --- | --- | --- | --- |
|  | r | p | | r | p | | r | p | |
| Corpus callosum genu | .05 | .24 | | .02 | .57 | | - | - | |
| L uncinate fasciculus | .18 | **.00003** | | .19 | **.00001** | | .05 | .29 | |
| R uncinate fasciculus | .06 | .15 | | .06 | .15 | | -.02 | .59 | |
| L anterior thalamic radiation | - | - | | - | - | | .08 | .07 | |
| R anterior thalamic radiation | - | - | | - | - | | .02 | .62 | |

Note: Uncorrected p-values for all correlations; L = left; R = right; Significant p-values are bolded; Blank cells denote that associations were not hypothesized a-priori; Exploratory results are reported in Supplementary Table 2.

Supplementary Table 2. Correlations between white-matter fractional anisotropy and personality traits (using the NEO-FFI measured at age 70: agreeableness, conscientiousness, and openness) for the exploratory analyses (false discovery rate (FDR) corrected p-values).

|  | Agreeableness | | Conscientiousness | | | Openness | | |  |
| --- | --- | --- | --- | --- | --- | --- | --- | --- | --- |
|  | r | p | | r | p | | r | p | |
| Corpus callosum genu | - | - | | - | - | | -.08 | 0.15 | |
| Corpus callosum splenium | .06 | 0.21 | | .11 | **0.05** | | .06 | 0.26 | |
| L anterior thalamic radiation | .08 | 0.15 | | .06 | 0.25 | | - | - | |
| R anterior thalamic radiation | .03 | 0.46 | | .08 | 0.15 | | - | **-** | |
| L cingulum | .09 | 0.13 | | .11 | **0.05** | | -.05 | 0.31 | |
| R cingulum | .03 | 0.47 | | .09 | 0.13 | | -.01 | 0.75 | |
| L arcuate fasciculus | .05 | 0.31 | | .16 | **0.001** | | -.07 | 0.16 | |
| R arcuate fasciculus | .03 | 0.54 | | .06 | 0.26 | | -.04 | 0.46 | |
| L inf. longitudinal fasciculus | .09 | 0.13 | | .16 | **0.009** | | .07 | 0.19 | |
| R inf. longitudinal fasciculus | .07 | 0.15 | | .19 | **0.0005** | | -.04 | 0.46 | |

Note: FDR corrected p-values for all correlations; L = left; R = right; inf. = inferior; significant p-values are bolded; Blank cells denote that associations were hypothesized a-priori: These results are reported in Supplementary Table 1.

Supplementary Table 3. Correlations between white-matter fractional anisotropy and personality traits (using the mean score of the NEO-FFI measured at age 70 and the IPIP 50-item Big Five scale measured at age 73: agreeableness, conscientiousness, and openness) for the hypothesized links.

|  | Agreeableness | | Conscientiousness | | | Openness | | |  |
| --- | --- | --- | --- | --- | --- | --- | --- | --- | --- |
|  | r | p | | r | p | | r | p | |
| Corpus callosum genu | .05 | .25 | | .03 | .52 | | - | - | |
| L uncinate fasciculus | .19 | **.000002** | | .20 | **.000006** | | .07 | .11 | |
| R uncinate fasciculus | .08 | .07 | | .09 | **.04** | | .01 | .78 | |
| L anterior thalamic radiation | - | - | | - | - | | .08 | .07 | |
| R anterior thalamic radiation | - | - | | - | - | | .06 | .17 | |

Note: Uncorrected p-values for all correlations; L = left; R = right; Significant p-values are bolded; Blank cells denote that associations were not hypothesized a-priori; Exploratory results are reported in Supplementary Table 4.

Supplementary Table 4. Correlations between white-matter fractional anisotropy and personality traits (using the mean score of the NEO-FFI measured at age 70 and the IPIP 50-item Big Five scale measured at age 73: agreeableness, conscientiousness, and openness) for the exploratory analyses (false discovery rate (FDR) corrected p-values).

|  | Agreeableness | | Conscientiousness | | | Openness | | |  |
| --- | --- | --- | --- | --- | --- | --- | --- | --- | --- |
|  | r | p | | r | p | | r | p | |
| Corpus callosum genu | - | - | | - | - | | -.06 | 0.24 | |
| Corpus callosum splenium | .10 | 0.06 | | .10 | 0.06 | | .05 | 0.27 | |
| L anterior thalamic radiation | .09 | 0.11 | | .07 | 0.21 | | - | - | |
| R anterior thalamic radiation | .04 | 0.46 | | .11 | **0.03** | | - | **-** | |
| L cingulum | .09 | 0.08 | | .14 | **0.007** | | -.02 | 0.71 | |
| R cingulum | .05 | 0.31 | | .13 | **0.01** | | .03 | 0.55 | |
| L arcuate fasciculus | .06 | 0.24 | | .17 | **0.0009** | | -.05 | 0.31 | |
| R arcuate fasciculus | .02 | 0.69 | | .07 | 0.19 | | -.02 | 0.66 | |
| L inf. longitudinal fasciculus | .12 | **0.02** | | .14 | **0.004** | | .08 | 0.14 | |
| R inf. longitudinal fasciculus | .09 | 0.09 | | .18 | **0.0002** | | -.03 | 0.55 | |

Note: FDR corrected p-values for all correlations; L = left; R = right; inf. = inferior; significant p-values are bolded; Blank cells denote that associations were hypothesized a-priori: These results are reported in Supplementary Table 3.

**Supplementary Text**

Analyses using NEO-FFI scales (measured at age 70) or the mean scores of the NEO-FFI traits (measured at age 70) and IPIP traits (measured at age 73) produced largely the same results as reported in the main text, with all of the key results replicated across analyses. The full results of these analyses are detailed below.

***NEO-FFI measured at age 70***

As predicted, we observed significant positive associations for left uncinate fasciculus FA with conscientiousness (r = .19, p = .00001), and left uncinate fasciculus FA with agreeableness (r = .18; p = .00003). In contrast with the results in the main text, we did not observe a significant association between right uncinate fasciculus FA and conscientiousness (r = .06; p = .15), nor for right anterior thalamic radiation FA and openness (r = .02; p = .62). Full output is presented in Supplementary Table 1.

Our exploratory analyses revealed significant associations between conscientiousness and FA measured in left cingulum (r = .11, p = .05), inferior longitudinal fasciculus (r = .16/.19; p = .009/.0005, L/R hemisphere, respectively), and left arcuate fasciculus (r =.16; p = .001). In contrast to results reported in the main text, we did not observe significant associations between right cingulum FA and conscientiousness (r = .09, p = .13), right anterior thalamic radiation and conscientiousness (r = .08; p = .15), or left inferior longitudinal fasciculus and agreeableness (r = .09; p = .13). However, an additional association was observed between corpus callosum splenium and conscientiousness (r = .11, p = .05). Full output is presented in Supplementary Table 2.

Left uncinate fasciculus (*β* = .16, p = .008) remained an independent predictor of conscientiousness when controlling for mean tract integrity (left uncinate fasciculus was correlated with mean tract integrity r = .62, p < .001). Moreover, this association was almost unchanged when additionally controlling for history of stroke, hypertension, and smoking. However, the other significant associations between FA and personality fell below nominal significance when controlling for mean tract integrity. Mean tract integrity was associated with agreeableness (r = .12, p = .005) and conscientiousness r = .19, p < .001, but not with openness r = .01, p = .91)

Analyses with the latent variable stability produced results that were closely matched to those reported in the main text (see Supplementary Figure 2).

***Mean Big Five Scores***

As predicted, we observed significant positive associations for uncinate fasciculus FA bilaterally with conscientiousness (r = .20/.09; p = .000006/.04, L/R hemisphere, respectively), and for left uncinate fasciculus FA with agreeableness (r = .19; p = .000002). The sole exception from results reported in the main text is that we did not observe a significant association between right anterior thalamic radiation FA and openness (r = .06; p = .17). Full output is presented in Supplementary Table 3.

Our exploratory analyses revealed significant associations between conscientiousness and FA measured in bilateral cingulum (r = .14/.13; p = .007/.001, L/R hemisphere, respectively), inferior longitudinal fasciculus (r = .14/.18; p = .004/.0002, L/R hemisphere, respectively), left arcuate fasciculus (r =.17; p = .0009), and right anterior thalamic radiation (r =.11; p = .003). Agreeableness was significantly associated with FA in left inferior longitudinal fasciculus (r =.12; p = .02). Full output is presented in Supplementary Table 4.

Left uncinate fasciculus (*β* = .15, p = .008) remained an independent predictor of conscientiousness when controlling for mean tract integrity (left uncinate fasciculus was correlated with mean tract integrity r = .62, p < .001). Moreover, this association was almost unchanged when additionally controlling for history of stroke, hypertension, and smoking. However, the other significant associations between FA and personality fell below nominal significance when controlling for mean tract integrity. Mean tract integrity was associated with agreeableness (r = .13, p = .001) and conscientiousness r = .22, p < .001, but not with openness r = .04, p = .34)

Analyses with the latent variable stability produced results that were closely matched to those reported in the main text (see Supplementary Figure 4).
